# Fluorescent Labeling of Inulin-Based siRNA Delivery Nanocarriers: Implications for Stability and Biological Performance

**DOI:** 10.1021/acsomega.6c02336

**Published:** 2026-06-04

**Authors:** Carmela Mazzacano, Gaia Scoppetta, Giulia Auriemma, Gaia Zanella, Gianluca Matteoli, Giovanni Falcone, Pasquale del Gaudio, Carla Sardo, Rita Patrizia Aquino

**Affiliations:** † Department of Pharmacy, 19028 University of Salerno, Via Giovanni Paolo II, Fisciano 84084, Italy; ‡ Laboratory of Mucosal Immunology, Department of Chronic Diseases and Metabolism (CHROMETA), 26657 KU Leuven, Herestraat 49, Leuven 3000, Belgium

## Abstract

Fluorescently labeled nanoparticles are often implicitly assumed to mimic the behavior of their unlabeled counterparts, despite potential physicochemical perturbations induced by probe conjugation. Herein, we systematically investigated the effect of fluorophore chemistry and labeling density on the colloidal stability and short interfering RNA (siRNA)-binding performance of inulin-based polymeric nanoparticles functionalized with branched polyethylenimine (bPEI) and poly­(D,l-lactic acid) (PLA). Two fluorophores, cyanine 7.5 (Cy7.5) and fluorescein isothiocyanate (FITC), were introduced via distinct conjugation strategies at variable grafting densities. Light scattering analyses revealed probe- and density-dependent modulation of nanoparticle size distribution and surface charge, with high FITC density inducing increased heterogeneity. Polyanion competition and RNase protection assays showed preserved siRNA stability for Cy7.5-labeled nanoparticles, whereas FITC conjugation reduced the level of siRNA retention at high labeling densities. Cellular uptake studies in MC38 cells demonstrated a clear overlap of fluorescent signals from Cy7.5 nanosystems and delivered siRNA under serum-free conditions, while serum proteins promoted partial siRNA displacement. Overall, these results demonstrate that fluorescent labeling is not a neutral modification and must be critically validated to avoid misinterpretation in fluorescence-based nanomedicine studies.

## Graphical abstract

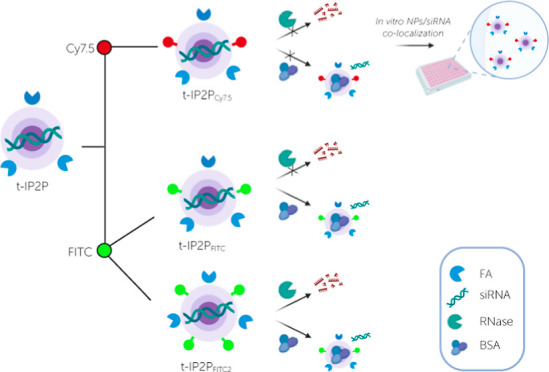

## 1 Introduction

Polymeric nanocarriers have been widely investigated as short interfering RNA (siRNA) carriers, owing to their ability to protect nucleic acids and promote cellular uptake.^1^ Among biocompatible materials, polymers offer the additional advantage of chemical versatility, enabling the conjugation of fluorescent probes that are commonly used to obtain fluorescent nanoparticles (NPs).^2^ Fluorescently labeled NPs have played a major role in bioimaging, live-cell analysis, biosensing, and theranostic applications, highlighting the broad utility of fluorescence-based tracking strategies in nanomedicine.^3^ Despite their widespread use, fluorescent labels are often assumed to not affect NP behavior. However, the chemical nature, charge, and density of the fluorophore may influence NP stability, surface properties, and interactions with biological components^4^ These considerations are especially relevant in the case of siRNA-loaded polymeric NPs, where even subtle structural modifications may affect the balance of forces governing NP assembly, siRNA retention, release, and protection. Indeed, changes introduced even in polymer regions not directly involved in nucleic acid complexation may influence nanocarrier performance when they perturb the hydrophilic/hydrophobic balance or introduce additional ionizable groups.^5,6^ To date, systematic studies evaluating the impact of fluorescent labeling on the performance of siRNA-loaded polymeric NPs remain limited, with many examples of works in which this modification is assumed to faithfully recapitulate the behavior of unlabeled NPs.^7–9^

Recent studies have raised important concerns regarding the interpretation of fluorescence-based experiments on nanostructured systems. The stability of labeled nanostructures under biologically relevant conditions has emerged as a critical parameter for the accurate interpretation of fluorescence-based experiments, as these systems may undergo degradation, disassembly, or interactions with biomolecules present in biofluids; such processes can alter the observed fluorescent signal, potentially leading to misinterpretation of the data if it is not verified that the structure remains intact throughout the experiment.^10^ In particular, Lacroix and co-workers demonstrated that fluorescent readouts from labeled DNA nanostructures may reflect the uptake of released dye rather than that of intact constructs, thereby generating potentially misleading intracellular localization data.^11^

At the same time, several studies on fluorescent siRNA delivery systems have highlighted the analytical value of probe-assisted tracking for following NP integrity, siRNA release, and cellular uptake, further underscoring the need to distinguish informative labeling from perturbing labeling.^12–15^

In this context, our inulin-based nanoplatform is particularly well suited to investigate the impact of fluorescent labeling because of its intrinsic modularity and structural controllability. Unlike less-defined systems, including many DNA-based constructs or conventional polymeric NPs, this platform allows systematic control over probe density and positioning while maintaining the same overall carrier architecture. Such a feature enables a more rigorous isolation of fluorophore-induced effects from other formulation-related variables and directly addresses the need for a model system in which structure–property relationships can be examined in a controlled manner.

Moreover, the use of two chemically distinct fluorophores, fluorescein isothiocyanate (FITC) and cyanine 7.5 (Cy7.5), provides a robust framework to investigate how probe chemistry affects NP behavior. Variations in fluorophore charge, hydrophobicity, and molecular structure may alter copolymer–siRNA interactions and, consequently, the physicochemical properties of the resulting NPs, with potential effects on their interactions with biological membranes and serum proteins. By comparing these probes within the same polymeric platform, we therefore assess whether fluorescent labeling can be considered a nonperturbing modification.

Taken together, this work is intended not only to clarify the specific case of fluorescently labeled inulin-based siRNA nanocarriers but also to propose a more comprehensive validation approach for traceable nanomedicines. On the basis of our results, we suggest that fluorescent labeling should not be treated as a simple auxiliary modification but should instead be assessed step by step throughout nanosystem development. Labeling-induced bias may emerge at different stages of characterization (whether the probe alters siRNA complexation, the colloidal properties of the nanosystem, or its stability against RNase degradation and displacement by competing biomolecules) and may become evident only at the final biological evaluation stage. This progressive framework provides a more reliable basis for the interpretation of fluorescence-based nanomedicine studies.

## 2 Experimental Section

### 2.1 Materials and Equipment

A complete list of materials and equipment is reported in Supporting Information (Paragraphs S1 and S2).

### 2.2 Synthesis and Characterization of Fluorescently Labeled Inulin-Based Copolymers

FITC-labeled copolymers, INU_FITC-bPEI2-PLA (FITC-IPA) and INU_FITC-bPEI2-PLA-FA (FITC-IPA-FA), were synthesized starting from FITC-labeled inulin (INU-FITC), by subsequent oxidation, conjugation of branched polyethylenimine (bPEI), poly­(D,l-lactic acid) (PLA), and finally folic acid (FA), following the same synthetic scheme previously reported.^5^ Detailed experimental procedures are reported in the Supporting Information (Paragraphs S3–S6). INU-bPEI2-PLA_Cy7.5 (Cy7.5-IPA) was synthesized by reacting INU-bPEI-PLA (IPA) with cyanine 7.5 NHS ester as detailed in Supporting Information (Paragraph S7). Fluorophore-labeled copolymers were characterized using ^1^H NMR, FTIR, and fluorescence spectroscopy as reported in Supporting Information (Paragraph S8). Finally, the relative buffering capacity of the copolymers was investigated through an acid–base titration (Paragraph S9).

### 2.3 siRNA-Loaded Nanoparticles Preparation

siRNA-loaded NPs were prepared at a copolymer/siRNA weight ratio (*R*) of 10 by simple mixing and incubation at room temperature. Full experimental details are reported in the Supporting Information (Paragraph S10). Different decorated NPs were obtained (Table 1), varying in the type of fluorescent probe (FITC or Cy7.5) and probe density. These formulations were prepared by mixing copolymers 1 and 2 at a 1:1 weight ratio, as detailed in Table 1.

**Table 1:** siRNA-Loaded Nanoparticle Composition

siRNA-loaded nanoparticle	copolymer 1	copolymer 2
t-IP2P	IPA-FA	IPA
t-IP2P_FITC_	IPA-FA	FITC-IPA
t-IP2P_FITC2_	FITC-IPA-FA	FITC-IPA
t-IP2P_Cy7.5_	IPA-FA	Cy7.5-IPA

### 2.4 siRNA-Loaded Nanoparticles Characterization

Agarose gel electrophoresis was employed to evaluate siRNA complexation (paragraph S11). NP hydrodynamic diameter and polydispersity index (PDI) were assessed by dynamic light scattering (DLS), while zeta potential (ZP) was measured using electrophoretic light scattering (Paragraph S12). The stability of complexes in the presence of polyanions was determined after incubation with bovine serum albumin (BSA) (Paragraph S13). The ability of complexes to protect siRNA from nuclease degradation was evaluated by incubation with RNase A (Paragraph S14). Cy7.5-labeled NPs were characterized in vitro in terms of cytocompatibility on MC38 and MODE-K cells (Paragraph S15) and uptake on MC38 cells (Paragraph S16).

## 3 Results and Discussion

### 3.1 Fluorescent Derivatives of IPA

Fluorescent derivatives of IPA were successfully obtained by conjugating FITC or Cy7.5 to the copolymer backbone through distinct chemical strategies, yielding systems characterized by different probe chemistries and substitutions (Figure 1).

**Figure 1:**
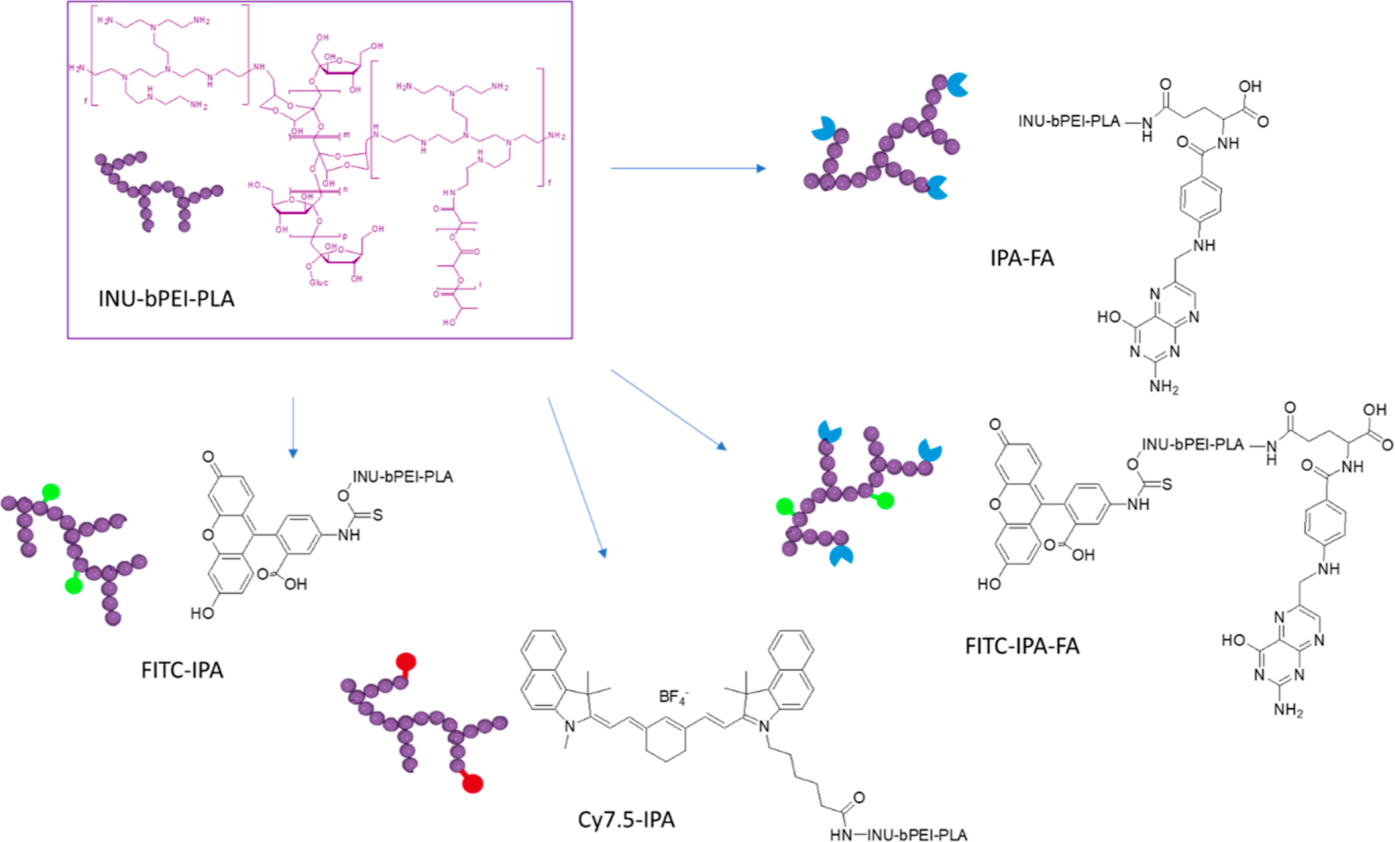
IPA and its fluorescent derivatives. Figure partially created in BioRender. Sardo, C. (2026) https://BioRender.com/4b83uhv.

The successful conjugation of FITC and Cy7.5 fluorescent probes to the copolymers was investigated by ^1^H NMR, FTIR, and fluorimetric analysis (Figure 2). Copolymers displayed characteristic aromatic resonances in the 6–9 ppm region, unambiguously confirming probe conjugation (Figure 2a,b). In the case of FITC-IPA-FA, the signals were partially overlapped to FA typical resonances in the same range (Figure 2b).

**Figure 2:**
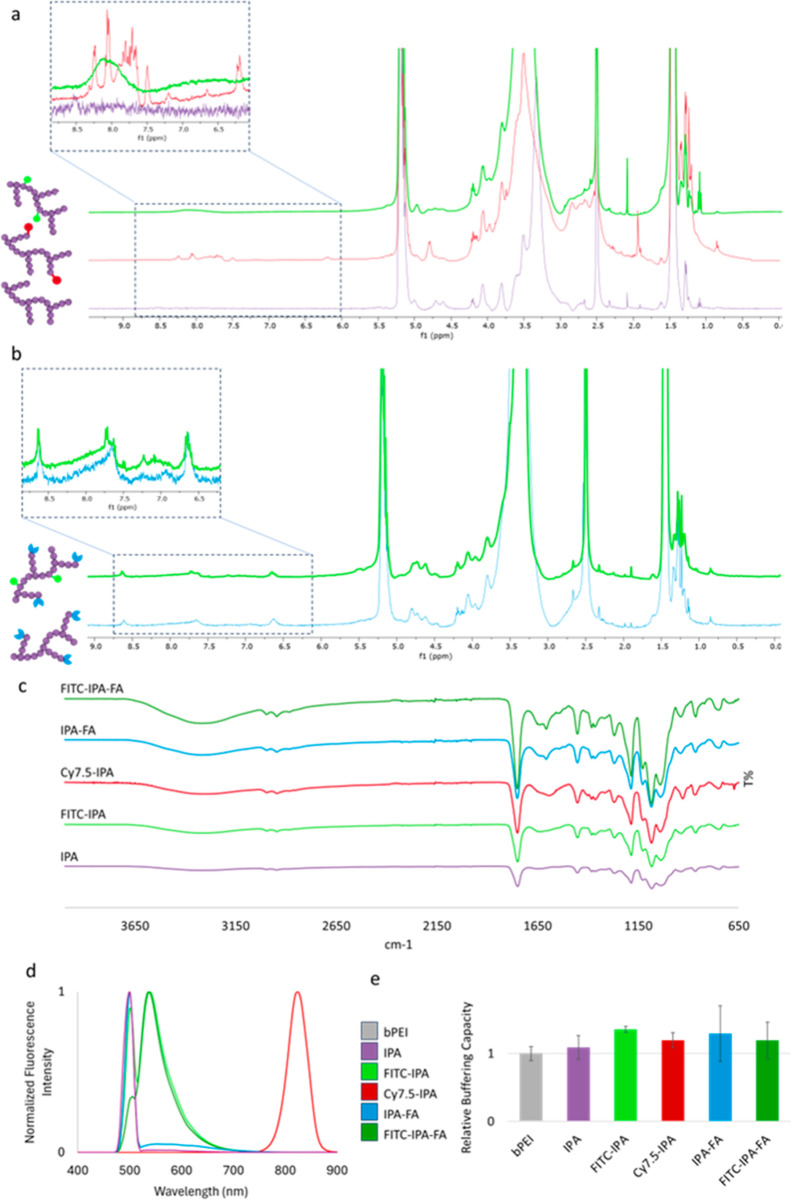
Characteristic ^1^H NMR spectrum of (a) IPA and its fluorescent derivatives, FITC-IPA and Cy7.5-IPA; (b) IPA-FA and its fluorescein-functionalized counterpart FITC-IPA-FA; (c) FTIR spectra of IPA and its fluorescent derivatives; (d) fluorescence emission spectra of FITC-IPA, FITC-IPA-FA, and Cy7.5-IPA; and (e) relative buffering capacity of copolymers. Figure partially created in BioRender. Sardo, C. (2026) https://BioRender.com/4b83uhv.

Fluorescence spectroscopy further validated the presence of the probes, revealing the expected emission maxima for FITC (∼495 nm) and Cy7.5 (∼800 nm) (Figure 2d). Notably, the excitation at 495 nm of IPA and IPA-FA gave an emission spectrum evidencing a peak at 500 nm, overlapping the shoulder that can be seen in the emission spectra of FITC-IPA and FITC-IPA-FA, compatible with the intrinsic fluorescence observed at cytofluorimetry and fluorescence microscopy.

To assess whether fluorophore conjugation affected the proton-buffering capacity of the bPEI domains, an essential feature for siRNA complexation and endosomal escape, acid–base titration studies were performed (Figure 2e). Cy7.5-IPA, FITC-IPA, and FITC-IPA-FA exhibited buffering profiles comparable to the unlabeled copolymers, indicating that fluorophore conjugation alone did not significantly impair the intrinsic buffering ability of bPEI (Welch’s *t*-test, *p* > 0.05). These findings indicate that whether the fluorophore is chosen for conjugation can be accommodated without disrupting proton buffering.

### 3.2 Fluorophore Chemistry and Density Differentially Affect siRNA Complexation and Colloidal Organization

The ability of fluorescently labeled copolymers to complex siRNA was first evaluated by agarose gel retardation assays (Figure 3A). Cy7.5-labeled nanoparticles (t-IP2P_Cy7.5_) retained siRNA with an efficiency comparable to that of unlabeled t-IP2P, indicating that Cy7.5 conjugation does not compromise the electrostatic complexation. Similarly, t-IP2P_FITC exhibited efficient siRNA binding at a low probe density. In contrast, increasing the FITC grafting density (t-IP2P_FITC2_) resulted in a marked reduction in siRNA retention, revealing a density-dependent destabilizing effect. Being anionic, FITC can interfere with the electrostatic interactions between the cationic segments of the copolymer and the anionic siRNA, which are essential for complex formation and stability. This effect becomes more pronounced at higher FITC grafting densities, ultimately leading to the partial destabilization of the copolymer–siRNA complex.

**Figure 3:**
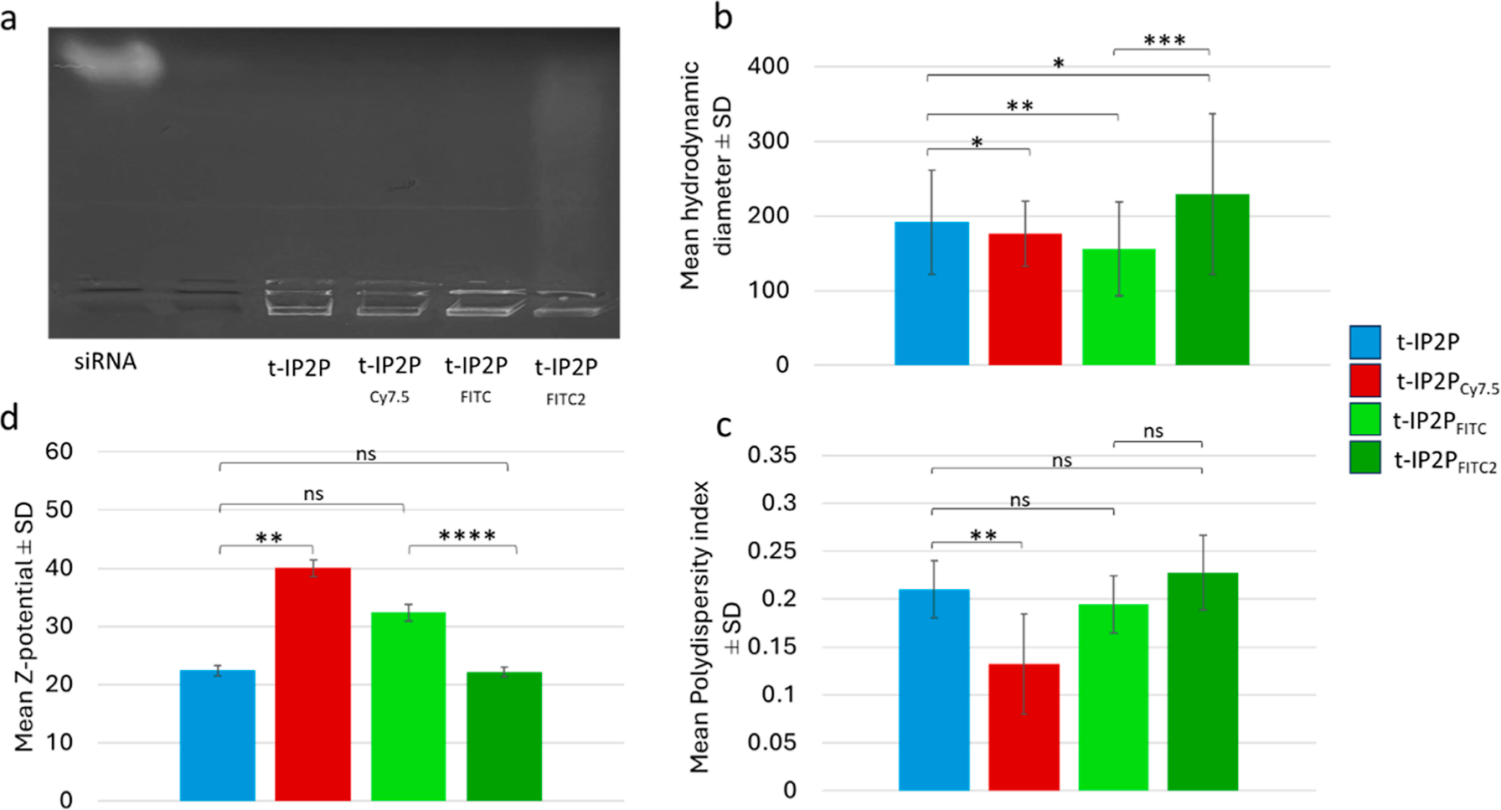
Agarose gel retardation assay (a) evaluating the siRNA complexation efficiency of unlabeled and fluorophore-labeled copolymers. Mean hydrodynamic diameter ±SD (b), polydispersity index (PDI) ± SD (c), and ZP ±SD (d) of siRNA-loaded t-IP2P, t-IP2P_Cy7.5,_ t-IP2P_FITC,_ and t-IP2P_FITC2_ NPs. Statistical analysis was performed using Welch’s *t*-test (*n* = 3) for particle size and ZP and Student’s *t*-test (*n* = 3) for PDI. Differences were considered statistically significant at *p < 0.05, **p < 0.01, ***p < 0.001, and ****p < 0.0001. A complete statistical comparison among all groups is provided in Table S1.

These differences were reflected in the colloidal properties of the resulting NPs. DLS analysis showed that Cy7.5 conjugation led to slightly smaller and more homogeneous NPs (176,35 nm ± 43,92 nm, PDI 0,13 ± 0,05) compared to the unlabeled system (191,81 nm ± 69,70 nm, PDI 0,21 ± 0,03) (Figure 3b,c). FITC-labeled NPs displayed a probe density-dependent behavior: low-density FITC conjugation produced smaller NPs (155,66 nm ± 62,81 nm) with relatively narrow size distributions (PDI 0,19 ± 0,03), whereas high FITC loading induced a substantial increase in particle size (224,41 nm ± 107,41 nm) and PDI (0,23 ± 0,04), indicative of enhanced heterogeneity and less controlled self-assembly.

ZP measurements further highlighted the impact of fluorophore chemistry on surface charge (Figure 3d). Cy7.5-labeled NPs exhibited an increased positive surface potential (40,05 mV ± 1,41 mV), whereas FITC-labeled systems showed intermediate or unchanged values (32,42 mV ± 1,43 mV for t-IP2P_FITC_ and 22,16 mV ± 0,86 mV for t-IP2P_FITC2_) compared to unlabeled NPs (22,38 mV ± 0,9 mV). The anionic nature of FITC likely counterbalances the positive charge of bPEI domains, particularly at higher labeling densities, thereby weakening electrostatic cohesion within the polyplex structure. While in other nanosystems anionic fluorophores may enhance colloidal stability via interparticle repulsion, in this case the dominant effect is the disruption of polymer–siRNA electrostatic interactions.

Collectively, these data indicate that fluorophore identity and loading density can significantly alter NP organization, even when the core polymer architecture remains unchanged.

### 3.3 Fluorescent Labeling Governs siRNA Stability under Biologically Relevant Destabilizing Conditions

Premature siRNA release triggered by competition with biologically relevant polyanions as well as enzymatic degradation by RNases represents two of the major barriers that nanosystems must overcome and are directly related to complex stability. These processes are critically governed by a delicate interplay of parameters such as NP architecture, charge density, and supramolecular organization, as previously reported.^5^ It is therefore essential to elucidate how Cy7.5- and FITC-labeled NPs preserve siRNA stability in the presence of BSA, employed here as a model polyanion, and under RNase challenge.

Notably, agarose gel electrophoresis of siRNA-loaded NPs after incubation with BSA showed that after 4 h t-IP2P_Cy7.5_ exhibited a behavior comparable to that of unlabeled t-IP2P, with no detectable siRNA release, demonstrating that Cy7.5 conjugation did not induce NP destabilization (Figure 4d). FITC-labeled systems displayed a different behavior: both t-IP2P_FITC_ and t-IP2P_FITC2_ showed siRNA displacement (Figure 4d), which was more pronounced for t-IP2P_FITC2_. This behavior may be attributed to the anionic nature of FITC, which could weaken the electrostatic interactions between siRNA and the cationic bPEI domains, thereby facilitating siRNA displacement in the presence of serum proteins such as albumin. Accordingly, the higher FITC content in t-IP2P_FITC2_ may further enhance the destabilizing effect.

**Figure 4:**
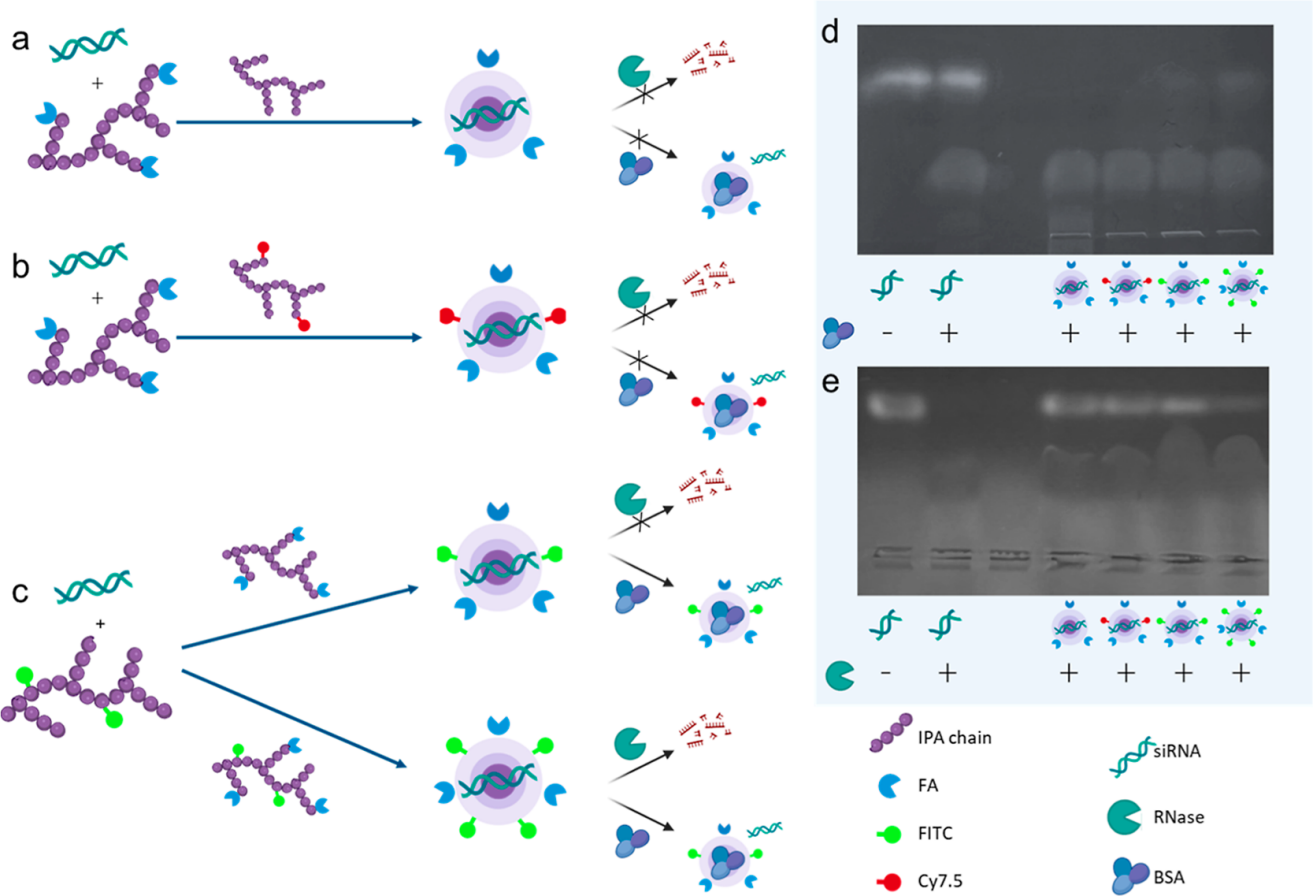
Agarose gel electrophoresis of siRNA-loaded NPs after incubation with BSA (panel d) or RNase A (panel e). (a) Unlabeled t-IP2P showed to be stable under both RNase and BSA; (b) Cy7.5 conjugation did not induce NP destabilization after RNase or BSA incubation; and (c) FITC conjugation at low density induced NP destabilization in the presence of BSA, while at high density it caused destabilization in the presence of both albumin and RNase. Created in BioRender. Sardo, C. (2026) https://BioRender.com/4b83uhv.

The ability of Cy7.5- and FITC-labeled NPs to retain the siRNA protection capability of the unlabeled NPs was evaluated by stability studies in the presence of RNase A. After 1 h of incubation with RNase A, siRNA integrity was assessed by agarose gel electrophoresis following its displacement from the NPs using SDS. As shown in Figure 4e, naked siRNA was completely degraded upon incubation with RNase A.^5^

Concerning the labeled systems, t-IP2P_FITC_ did not exhibit any significant alteration in siRNA protection capability compared to t-IP2P, whereas a reduced siRNA protection was detected for t-IP2P_FITC2_, confirming that FITC density plays a critical role in modulating NP stability (Figure 4e). In addition, Cy7.5 conjugation did not affect NP stability, as t-IP2P_Cy7.5_ showed siRNA protection capability from RNase degradation (Figure 4e).

### 3.4 Stability of t-IP2P_Cy7.5_ NPs in Biologically Relevant Environment In Vitro

On the basis of their superior colloidal and stability profiles, Cy7.5-labeled NPs were further evaluated in vitro. Cytocompatibility studies in MC38 and MODE-K cells revealed no significant differences between t-IP2P and t-IP2P_Cy7.5_ after 48 h of incubation (*p* > 0.05, Welch *t*-test, *n* = 3), indicating that Cy7.5 conjugation does not introduce additional cytotoxicity (Figure 5a,b).

**Figure 5:**
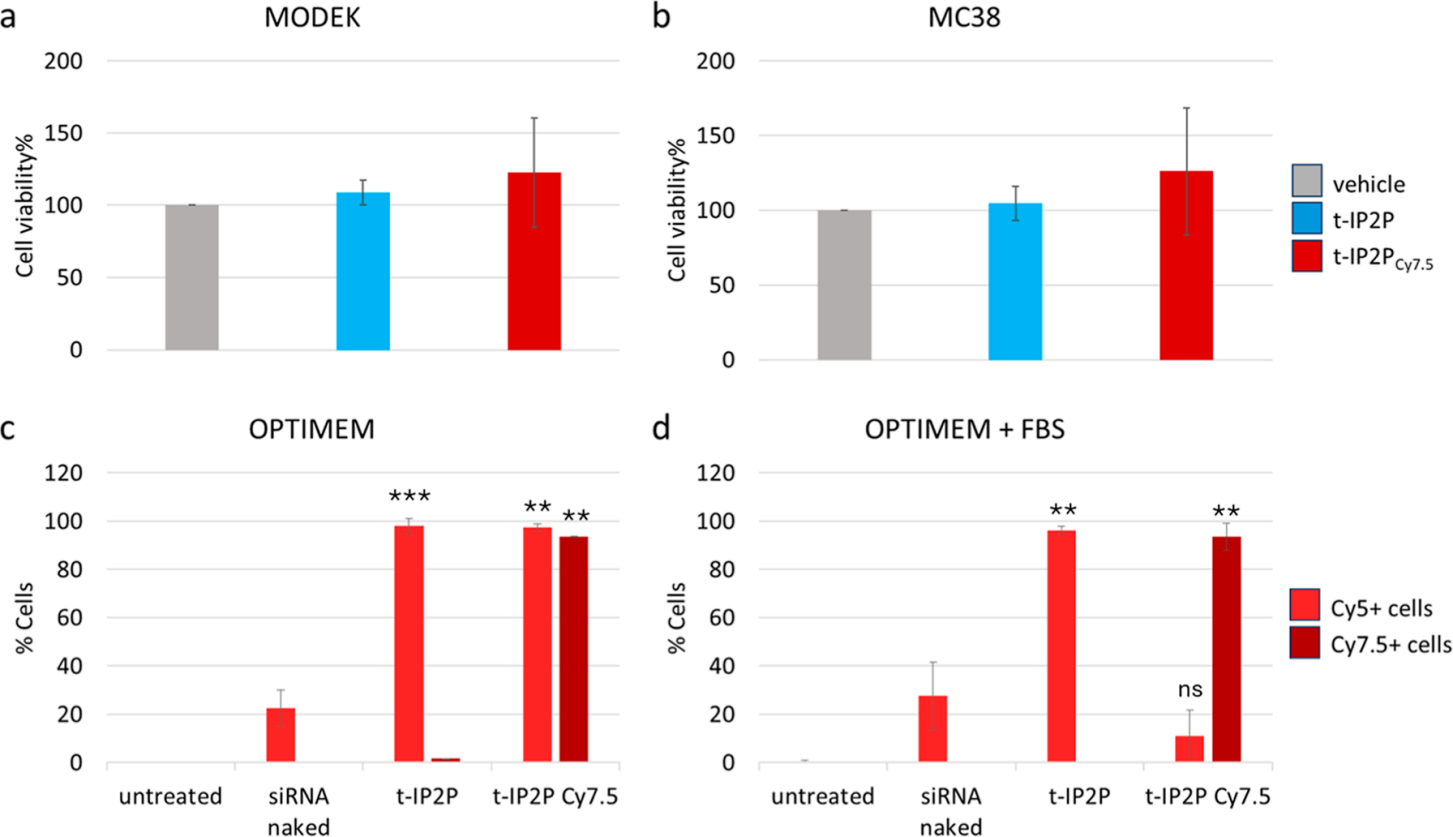
Cell viability of MC38 (a) and MODE-K (b) after 48 h of incubation with t-IP2P or t-IP2P_Cy7.5_ NPs. Cellular uptake of t-IP2P and t-IP2P_Cy7.5_ NPs loaded with Cy5-labeled siRNA in MC38 cells, evaluated by flow cytometry after 6 h of incubation in medium without FBS (c) and with FBS (d). Statistical analysis was performed using Welch’s *t*-test (*n* = 3). Differences were considered significant at *p < 0.05, **p < 0.01, and ***p < 0.001 (ns, *p* > 0.05). t-IP2P- and t-IP2P_Cy7.5_-treated groups were compared to the naked siRNA treatment. For statistical comparison between all groups, please refer to Table S2.

Cellular uptake of t-IP2P and t-IP2P_Cy7.5_ NPs loaded with a model Cy5-siRNA in MC38 cells was evaluated by flow cytometry after 6 h of incubation. As reported in Figure 5, the conjugation of Cy7.5 did not alter the siRNA uptake profile, as comparable levels of Cy5-siRNA-positive cells % were observed for both t-IP2P (97,93 ± 3,32) and t-IP2P_Cy7.5_ NPs (97,40 ± 1,51) (*p* > 0.05, Welch *t*-test, n = 3). Notably, a clear overlap of fluorescence signals from labeled nanoparticles and delivered siRNA was observed at the cellular level, as the percentage of cells positive for Cy5-siRNA closely matched those positive for the Cy7.5 signal (Figure 5c). However, this observation does not necessarily imply true intracellular colocalization within specific subcellular compartments. Demonstrating such colocalization would require a more detailed investigation of intracellular trafficking, for instance through fluorescence confocal microscopy or other high-resolution imaging techniques.

However, when uptake experiments were performed in the presence of serum, a marked reduction in Cy5-siRNA-positive cells % was observed for t-IP2P_Cy7.5_ (10,91 ± 5,98) (*p* < 0.01, Welch *t*-test, *n* = 3), while t-IP2P remained comparable (96,23 ± 1,62) (Figure 5d). This divergence suggests partial siRNA displacement in protein-rich environments, highlighting how biologically relevant conditions can unmask subtle destabilization effects that are not apparent under simplified experimental settings. These observations reinforce the importance of validating fluorescent labeling strategies under conditions that closely mimic the intended biological application.

## 4 Conclusions

In this study, inulin-based t-IP2P NPs were successfully functionalized with Cy7.5 and FITC fluorescent probes to obtain traceable siRNA delivery systems and to investigate the impact of fluorophore conjugation on NP performance. The results demonstrated that the effect of fluorescent labeling is strongly dependent on the nature and density of the probe. Cy7.5 conjugation preserved NPs siRNA protection capability and cytocompatibility, while enabling direct evaluation of NPs siRNA co-occurrence and cellular uptake. Moreover, uptake studies under biologically relevant conditions indicated that serum proteins may influence siRNA stability within Cy7.5-labeled NPs. Overall, this work provides useful insights into the rational design of fluorescently traceable polymeric nanocarriers, emphasizing the need to balance imaging capability with structural integrity and biological performance for an effective siRNA delivery.

## Supplementary Material



## Notes

Complete materials and equipment lists; detailed copolymers synthesis protocols; buffering assay protocol; detailed nanoparticles; and chemicophysical and in vitro characterization methods (PDF)
