# Supplementary material for: Fluorescent Labeling of Inulin-Based siRNA Delivery Nanocarriers: Implications for Stability and Biological Performance
Source: ACS Omega. 2026 Jun 4;11(23):34385–91. doi: 10.1021/acsomega.6c02336 (PMC13280891; doi:10.1021/acsomega.6c02336)
Supplement: Supplementary file 1 [file ao6c02336_si_001.pdf]

Supporting information to “Fluorescent Labeling of Inulin-based siRNA Delivery Nanocarriers: Implications for Stability and Biological Performance”

*Carmela Mazzacano[a ‡], Gaia Scoppetta[a ‡], Giulia Auriemma[a], Gaia Zanella[b], Gianluca Matteoli[b], Giovanni Falcone[a], Pasquale del Gaudio[a], Carla Sardo[a *], Rita patrizia Aquino[a]*

[a] Department of Pharmacy, University of Salerno, Via Giovanni Paolo II, Fisciano, 84084, Salerno, Italy

[b] Laboratory of Mucosal Immunology, Department of Chronic Diseases and Metabolism (CHROMETA), KU Leuven, Herestraat 49, Leuven, Belgium

*Corresponding author, csardo@unisa.it

‡ These authors contributed equally to this work

S1. Materials

Inulin from Dahlia tubers, with a molecular weight of approximately 5000 Da (INU), was procured from Merck, Milan. Prior to its application in reactions, it was desiccated at 70 °C overnight in an oven and cooled under vacuum in the presence of phosphorus pentoxide. Fluorescein isothiocyanate–inulin , with a molecular weight of approximately 3000-5000 Da (INU_FITC), was procured from Merck, Milan. Cyanine7.5 NHS ester was purchased from Lumiprobe Corporation, USA. The membranes employed for dialysis (Spectra/Por® MWCO 1 kDa and Spectra/Por® MWCO 3.5 kDa) were purchased from Spectrum Laboratories, Inc. Sodium metaperiodate (NaIO4), deuterated dimethyl sulfoxide (DMSO-d6), bPEI (Mw= 800 Da), deuterium oxide (D2O), dimethyl sulfoxide (DMSO), N,N-Dimethylformamide (DMF), N-(3-dimethylaminopropyl)-N′-ethylcarbodiimide hydrochloride (EDC HCl), N-hydroxysuccinimide (NHS), sodium cyanoborohydride (NaBH3CN), Folic Acid (FA), Acetic acid, Tris(hydroxymethyl)amino-methane, Sodium hydroxide, Sodium phosphate monobasic dihydrate, Potassium phosphate monobasic and Potassium chloride were obtained from Merck. PLA with an average molecular weight of 10000 Da was procured from PolySciTech® (Division of Akina, West Lafayette, IN). Diethyl ether (Et2O) was acquired from Carlo Erba Reagents. Acetone was obtained from Clean Consult International S.p.A. (CCI). Agarose, ethidium bromide, RNase-free water, glucose, 20% albumin solution, Ribonuclease A, Sodium chloride and the equipment for gel electrophoresis were from Carl Roth. Tert-butyl carbazate (TBC) was procured from Purac Biochem B.V. Propylene glycol was obtained by Galeno srl. EDTA disodium salt dihydrate was purchased from ITW Reagents. Hydrochloric acid was from Honeywell. Sodium laurylsulphate (SDS) was purchased from A.C.E.F. spa. 3-(4,5-Dimethyl-2-thiazolyl)-2,5-diphenyl-2H-tetrazolium Bromide (MTT) was from Gerbu Biotechnik GmbH. High glucose Dulbecco's

Modified Eagle Medium (DMEM), Opti-MEM™ Reduced Serum Medium (OPTIMEM), Penicillin, Streptomycin, L- glutamine, N-2- hydroxyethylpiperazine-N-2-ethane sulfonic acid (HEPES), 2-Mercaptoethanol and 1X Non-Essential Amino Acids were from Gibco, ThermoFisher Scientific. Fetal Bovine Serum (FBS) was from Biowest.For nanosystems preparation and technological characterization MISSION® siRNA Universal Negative Control #1 from Merck, Milan was used. For in vitro biological characterization studies MISSION® siRNA Fluorescent Universal Negative Control #1, Cyanine 5 from Merck (siSCR-Cy5) was used.

S2. Equipment

1H NMR spectra were recorded using a Bruker Ascend 400 nuclear magnetic resonance spectrometer operating at a frequency of 400 MHz. FT-IR spectra were acquired using a Frontier FT-IR spectrometer (Perkin Helmer) equipped with a single reflection ATR sampling module. Freeze drying was made on a BUCHI Lyovapor L-200 apparatus. DLS and ELS experiments were conducted using an Anton Paar Litesizer 500 instrument with a 658 nm laser at a fixed scattering angle of 175°. Lab Solutions Lite software (Shimadzu, Kyoto, Japan) was utilized for data acquisition. pH measurements were made on a Mettler Toledo Seven Compact S210 pHmeter. Fluorescence spectra were recorded using a SHIMADZU RF-6000 spectrophotofluorometer. Thermo Scientific Heraeus Labofuge 200 Centrifuge was used for centrifugation procedures. UV-Vis measurements were performed on FLUOstar Omega plate reader, BMG LABTECH. For flow cytometry analysis a Sony SA3800 Spectral Cell Analyzer (Sony Biotechnology Inc. San Jose, California) was used.

S3. Synthesis of Oxidized Inulin Derivatives INU-OX and INU_FITC-OX

Briefly, INU or INU_FITC (1 g, 6.167 mmol of fructose repeating units) was dissolved in 8 mL of double-distilled water at 60 °C. After cooling to room temperature, $NaIO_4$ (0.075 equiv per fructose repeating unit) was added and the mixture was stirred in the dark at 40 °C for 5 h. The reaction was quenched by addition of excess propylene glycol (propylene glycol/$NaIO_4$ molar ratio = 3), and the mixture was dialyzed (MWCO 1 kDa) against double-distilled water. The purified oxidized products, INU-OX or INU_FITC-OX, were obtained by freeze-drying. The number of reactive aldehydes on INU-OX and INU_FITC-OX derivatives, expressed as millimoles of reactive aldehyde per 100 mmol of INU repeating units (DDCHO mol%), was determined through 1H NMR after functionalization of the products with TBC [1].

S4. Synthesis of INU-bPEI2 and INU_FITC-bPEI2

INU-OX or INU_FITC-OX (250 mg) was dissolved in 5 mL of double-distilled water and added dropwise to an aqueous solution of bPEI2 (10 equiv per INU-OX aldehyde group) under constant stirring at room temperature. The pH was adjusted to 5 using 5 M HCl and $NaBH_3CN$ (1.1 equiv per bPEI) was added and the mixture was stirred for 24 h at 30 °C. The resulting product was purified by exhaustive dialysis (MWCO 3.5 kDa) against double-distilled water and isolated by freeze-drying, yielding INU-bPEI2 or INU_FITC-bPEI2, respectively. For FITC-containing derivatives (INU_FITC-bPEI2), both the reaction and the subsequent dialysis steps were performed in the dark in order to preserve the photophysical integrity of the fluorescent probe. The obtained products were characterized by 1H NMR in D2O and by FT-IR.

S5. Synthesis of INU-bPEI2-PLA and INU_FITC-bPEI2-PLA

PLA (1.5 equiv per mmol of INU-bPEI2) was dissolved in 0.6 mL of DMSO and NHS (5 equiv per mmol PLA) and EDC (5 equiv per mmol PLA), previously dissolved in 0.45 mL of DMSO,

were sequentially added and the mixture was stirred for 2 h at room temperature. The activated PLA solution was then added dropwise to a solution of INU-bPEI2 or INU_FITC-bPEI2 in DMSO (34.5 mg/mL) and stirred for 24 h at room temperature. The resulting copolymers were precipitated in 40 mL of cold diethyl ether/acetone (85:15, v/v) and collected by centrifugation (5000 rpm, 10 min). The sediment was washed three times with 40 mL of the same solvent mixture and dried under reduced pressure. The solid was then resuspended in water and further purified by three washing cycles followed by freeze-drying, yielding INU-bPEI2-PLA or INU_FITC-bPEI2-PLA. For FITC-labeled copolymers (INU_FITC-bPEI2-PLA), all reaction and purification steps, including precipitation and dialysis, were carried out in the dark to prevent photobleaching of the fluorophore. The obtained products were characterized by 1H NMR in DMSO-d6 and by FT-IR.

S6. Synthesis of INU-bPEI2-PLA-FA and INU_FITC-bPEI2-PLA-FA

Folic acid (FA, 3 equiv per mmol of INU-bPEI2-PLA or INU_FITC-bPEI2-PLA) was dissolved in 0.6 mL of DMSO. NHS (5 equiv per mmol FA) and EDC (5 equiv per mmol FA), previously dissolved in 0.45 mL of DMSO, were successively added, and the mixture was stirred for 2 h at room temperature to activate the FA.

The activated FA solution was added dropwise to INU-bPEI2-PLA or INU_FITC-bPEI2-PLA (50 mg in 1.45 mL of DMSO) under constant stirring. The reaction was allowed to proceed for 24 h at room temperature. The reaction was quenched by the addition of 10 mL of water, and the pH was adjusted to 7 using 0.2 M NaOH. The product was purified by exhaustive dialysis (MWCO 1 kDa) against double-distilled water and recovered by freeze-drying as a yellow solid, yielding INU-bPEI2-PLA-FA or INU_FITC-bPEI2-PLA-FA. For FITC-containing samples, all reaction and

purification steps were performed in the dark to preserve the fluorescent probe. The obtained products were characterized by 1H NMR in DMSO-d6 and by FT-IR.

S7. Post-Synthetic Fluorescent Labeling of INU-bPEI2-PLA with Cyanine 7.5

INU-bPEI2-PLA (25 mg) was dissolved in 650 μL of anhydrous DMF. Separately, Cyanine 7.5 NHS ester (1 mg) was dissolved in 470 μL of anhydrous DMF and added dropwise to the copolymer under continuous stirring in a nitrogen atmosphere. The reaction was carried out for 24 h at room temperature in the dark. After completion of the reaction, an additional 25 mg of INU-bPEI2-PLA was added and the product was purified by exhaustive dialysis (MWCO 1 kDa) against double-distilled water in the dark and isolated by freeze-drying. The obtained products were characterized by 1H NMR in DMSO-d6 and by FT-IR.

S8. Buffering Capacity

Copolymer samples were prepared by suspending 6 mg in 30 mL of double-distilled water (0.2 mg/mL). The pH of the solution was initially adjusted to 10 using 0.1 N NaOH. Titration was performed by gradual addition of 30 μL aliquots of 0.1 M HCl until the pH reached 3. For comparison, free bPEI at the same concentration as in the copolymer was titrated.

The buffering capacity in the pH range 7.4–5.1 ($BC_{7.4–5.1}$) was calculated according to the equation:

$$BC_{7.4\text{-}5.1} = (V_{HCl} \cdot 0.1\ \text{M}) \cdot 100/\text{bPEImol}$$

where V_{HCl} is the volume in L of 0.1 M HCl used to adjust the pH values of the solution from 7.4 to 5.1, as determined by curve fitting with a sigmoid, and bPEImol is the total moles of bPEI in

the analysed solution. The relative buffering capacity (BC_{Rel}) was calculated by comparing the BC of the copolymer with the BC of the bPEI, determined utilizing a solution containing bPEI at the same concentration as in the copolymer:

$$BC_{Rel} = BC_{7.4\text{-}5.1}\ \text{Copolymer} / BC_{7.4\text{-}5.1}\ \text{bPEI}$$

S9. siRNA loaded nanoparticles characterization

A total of 30 mg of copolymers, mixed at a 1:1 weight ratio, were dissolved in 1 mL of DMF. Specifically, t-IP2P nanoparticles were prepared using INU-bPEI2-PLA and INU-bPEI2-PLA-FA mix, t-IP2PFITC using INU_FITC-bPEI2-PLA and INU-bPEI2-PLA-FA mix, t-IP2PFITC2 using INU_FITC-bPEI2-PLA and INU_FITC-bPEI2-PLA-FA mix and finally t-IP2PCy7.5 using INU-bPEI2-PLA_Cy7.5 and INU-bPEI2-PLA-FA. Subsequently, 9 mL of RNase-free water were slowly added under vigorous stirring. The resulting dispersions were extensively dialyzed against RNase-free water (MWCO 10,000 Da, CE, Float-A-Lyzer®), with water changes every 2 h, and finally lyophilized. siRNA loaded NPs were then prepared in RNase-free distilled water by mixing a fixed volume of lyophilized copolymers mix aqueous dispersion (1 mg/mL) with an equal volume of siRNA aqueous solution (0.1 mg/mL), achieving a copolymer/siRNA weight ratio (R) of 10. The mixtures were gently pipetted up and down ten times and incubated at room temperature for 1 h. For cellular studies, lyophilized copolymers mix aqueous dispersion were prepared using sterile RNase-free water and filtered through a 0.22 µm membrane, siRNA aqueous solution was prepared using sterile RNase-free water and copolymer to siRNA mix was made under laminar flow hood.

S10. Agarose gel electrophoresis

Agarose gel electrophoresis was employed to evaluate siRNA complexation. 5 µL of a glucose solution (250 mg mL^{-1}) were added to 20 µL of t-IP2P, t-IP2P$_{Cy7.5}$, t-IP2P$_{FITC}$ and t-IP2P$_{FITC2}$ NPs containing 1 µg of siRNA, prepared at R10. Each sample was loaded onto a 1.5% (w/v) agarose gel prepared in nuclease-free Tris–acetate–EDTA (TAE) buffer (0.4 M Tris acetate, 0.01 M EDTA, pH 8.3) supplemented with ethidium bromide (5 µg mL^{-1}). Electrophoresis was carried out at 80 V for 30 min. Gels were visualized under UV using a transilluminator and documented by digital camera.

S11. Measurement of Nanoparticles Sizes and Zeta Potential

Dynamic light scattering (DLS) measurements were performed at 25 °C to determine the intensity-weighted hydrodynamic diameter (nm) and polydispersity index (PDI) by cumulative analysis of the autocorrelation function. Zeta potential (ZP) was measured by electrophoretic light scattering (ELS) using the same instrument, and values were calculated from electrophoretic mobility according to the Smoluchowski model. DLS analyses were carried out on 50 µL of t-IP2P, t-IP2P$_{Cy7.5}$, t-IP2P$_{FITC}$ and t-IP2P$_{FITC2}$ NPs containing 1 µg of siRNA, prepared at R10. For ELS measurements, samples were diluted 1:12 in nuclease-free water. All measurements were performed in triplicate.

S12. Polyanion competition assay

t-IP2P, t-IP2P$_{Cy7.5}$, t-IP2P$_{FITC}$ and t-IP2P$_{FITC2}$ NPs containing 1 µg of siRNA, prepared at R10 (20 µL), were mixed with 5 µL of a bovine serum albumin (BSA) solution at 200 mg mL^{-1} by gentle

pipetting and incubated at room temperature for 4 h. Agarose gel electrophoresis was subsequently performed as described above.

S13. RNAse protection assay

20 µL t-IP2P, t-IP2P$_{Cy7.5,}$ t-IP2P$_{FITC}$ and t-IP2P$_{FITC2}$ NPs containing 1 µg of siRNA were incubated at 37 °C for 1 h with 3 µL of RNase A solution in DPBS (pH 7.4), corresponding to 5 mIU of RNase A per µg of siRNA. After incubation, RNase A was inactivated by heating the samples at 70 °C for 30 min. Subsequently, siRNA was displaced from the NPs by incubation with 3 µL of a 2% (w/v) SDS solution at room temperature for 10 min. Agarose gel electrophoresis was then performed as described above to evaluate siRNA integrity.

S14. In vitro cell viability

Murine colon adenocarcinoma cell line MC38 (NCI, ENH204-FP) was kindly provided by Prof. Max Mazzone (VIB - KU Leuven -, Belgium). The MC38 cell line was maintained in 5 % CO2 at 37 °C in high glucose DMEM supplemented with 10 % FBS, 100 µg/mL Penicillin and Streptomycin, 2 mM L-glutamine, 10 mM HEPES, 1 mM sodium pyruvate, 50 µM 2-Mercaptoethanol and 1X Non-Essential Amino Acids.

Mouse MODE-K Intestinal Epithelial cell line MODE-K was kindly provided by Prof. Gianluca Matteoli (Targid, KU Leuven, Belgium). The MODE-K cell line was maintained in 5 % CO2 at 37 °C in high glucose DMEM supplemented with 10 % FBS, 100 µg/mL Penicillin and Streptomycin.

MC38 or MODE-K cells (5 × 10^3 cells/well) were seeded in 96-well flat-bottom plates and incubated for 24 h at 37 °C in a humidified atmosphere containing 5% CO_2. On day 1, when cell

confluence reached approximately 30–40%, cells were washed twice with sterile PBS and then exposed to t-IP2P or t-IP2P$_{Cy7.5}$ NPs prepared as described above and diluted 1:20 in OPTIMEM. After 48 h of incubation, the medium containing the NPs was removed and 200 μL of MTT solution (0.5 mg/mL in DMEM) was added to each well. Following 3 h of incubation, the MTT solution was removed and 100 μL per well of isopropanol containing 0.1 M HCl was added to solubilize the formazan crystals. The plate was incubated at room temperature under gentle agitation for 30 min in the dark, and absorbance was measured at 560 nm using a microplate reader. Each experiment was conducted in triplicate.

S15. Cellular uptake

5 x 10^4 MC38 cells were cultured into a 24-well glass slide and incubated for 24 hours at 37°C in 5% humidified CO2. On day 1, the cells were exposed to siRNA-Cy5 loaded t-IP2P or t-IP2P$_{Cy7.5}$ NPs in OPTIMEM in the absence and in the presence of 10% FBS. After 6 hours of incubation, the cells were collected, washed, suspended in PBS and analyzed with a Sony SA3800 Spectral Cell Analyzer (Sony Biotechnology Inc. San Jose, California) flow cytometry apparatus. Each experiment was conducted in triplicate.

Table S1. Statistical analysis of DLS and ELS using Welch's t-test for particle size and zeta potential, and Student's t-test for PDI (*p < 0.05, **p < 0.01, ***p < 0.001, and ****p < 0.0001).

	p value<br>size		p value<br>PDI		p value<br>Z-pot	
t-IP2P vs t-IP2P$_{Cy7.5}$	0.029475293	*	0.00173578	**	0.005588198	**
t-IP2P vs t-IP2P$_{FITC}$	0.004415641	**	0.25010911	ns	0.099994897	ns
t-IP2P vs t-IP2P$_{FITC2}$	0.037485463	*	0.38576686	ns	0.276279062	ns
t-IP2P$_{Cy7.5}$ vs t-IP2P$_{FITC}$	0.050642247	ns	0.01009498	*	0.005867549	**
t-IP2P$_{Cy7.5}$ vs t-IP2P$_{FITC2}$	0.006780894	**	0.00080049	***	1.78467E-05	****
t-IP2P$_{FITC}$ vs t-IP2P$_{FITC2}$	0.000849145	***	0.07451689	ns	7.27975E-07	****

Table S2. Statistical analysis of cellular uptake by Welch t-test (*p<0.05, **p<0.01, ***p<0.001,****p<0.0001).

	Medium	Cy5+ cells	Cy7.5+ cells
		**p value**	**p value**
untreated vs siRNA naked	OPTIMEM	0,035879835	0,438027
untreated vs tIP2P	OPTIMEM	0,000373305	0,095692
untreated vs $tIP2P_{Cy7.5}$	OPTIMEM	6,67727E-05	0,00297
siRNA naked vs tIP2P	OPTIMEM	0,000881849	0,100381
siRNA naked vs $tIP2P_{Cy7.5}$	OPTIMEM	0,002481488	0,002971
tIP2P vs $tIP2P_{Cy7.5}$	OPTIMEM	0,817732603	0,002828
untreated vs siRNA naked	OPTIMEM + FBS	0,077168729	0,475839434
untreated vs tIP2P	OPTIMEM + FBS	6,71664E-05	0,227736059
untreated vs $tIP2P_{Cy7.5}$	OPTIMEM + FBS	0,244941554	0,001204996
siRNA naked vs tIP2P	OPTIMEM + FBS	0,012488452	0,425091189
siRNA naked vs $tIP2P_{Cy7.5}$	OPTIMEM + FBS	0,179366828	0,001211808
tIP2P vs $tIP2P_{Cy7.5}$	OPTIMEM + FBS	0,004736092	0,001211035
tIP2P vs tIP2P	OPTIMEM and OPTIMEM + FBS	0,485793436	0,115607
$tIP2P_{Cy7.5}$ vs $tIP2P_{Cy7.5}$	OPTIMEM and OPTIMEM + FBS	0,004690983	0,995925

Table S3. List of Abbreviations

Abbreviation	Full term
INU	Inulin
bPEI	Branched Polyethylenimine
PLA	Poly(D,L-lactic acid)
FA	Folic Acid
IPA	INU-bPEI-PLA copolymer
FITC	Fluorescein Isothiocyanate
Cy7.5	Cyanine 7.5
siRNA	Small Interfering RNA
NPs	Nanoparticles

Table S3. List of Abbreviations	
**Abbreviation**	**Full term**
DLS	Dynamic Light Scattering
ELS	Electrophoretic Light Scattering
PDI	Polydispersity Index
ZP	Zeta Potential
BSA	Bovine Serum Albumin
RNase A	Ribonuclease A
SDS	Sodium Dodecyl Sulfate
NMR	Nuclear Magnetic Resonance
FTIR	Fourier Transform Infrared Spectroscopy
MC38	Murine Colon Carcinoma Cell Line
MODE-K	Murine Intestinal Epithelial Cell Line
FBS	Fetal Bovine Serum
Cy5	Cyanine 5
NHS	N-hydroxysuccinimide
R	Copolymer/siRNA weight ratio
t-IP2P	Target nanoparticle formulation
t-IP2PFITC	FITC-labeled nanoparticle
t-IP2PFITC2	FITC-labeled nanoparticle (high density)
t-IP2PCy7.5	Cy7.5-labeled nanoparticle
CAC	Critical Aggregation Concentration
PBS	Phosphate Buffered Saline

Bibliography

(1) Mazzacano, C.; Auriemma, G.; Sellitto, M. R.; De Simone, V.; van Baarle, L.; Zanella, G.; Donnarumma, C.; Del Gaudio, P.; Sommella, E. M.; Golino, V.; La Gioia, D.; Merciai, F.; Campiglia, P.; Matteoli, G.; Sardo, C.; Aquino, R. P. Engineering a Library of Inulin Based Polyplexes and Core-Shell Nanoparticles: Inside the Targeted siRNA Delivery to Colorectal Cancer Cells. Materials Today Advances 2025, 28, 100645. https://doi.org/10.1016/j.mtadv.2025.100645.
